# Empagliflozin improves insulin sensitivity in patients with recent acute coronary syndrome and newly detected dysglycaemia: Experiences from the randomized, controlled SOCOGAMI trial

**DOI:** 10.1186/s12933-023-01950-0

**Published:** 2023-08-11

**Authors:** Elena Fortin, Magnus Lundin, Linda Mellbin, Anna Norhammar, Per Näsman, Stina Smetana, Peder Sörensson, Ele Ferrannini, Lars Rydén, Giulia Ferrannini

**Affiliations:** 1 Department of Medicine Solna, Karolinska Institutet, Stockholm, Sweden; 2 Department of Clinical Physiology, Karolinska Institutet, Stockholm, Sweden; 3 Cardiology Unit, Karolinska University Hospital, Stockholm, Sweden; 4 Capio S:t Görans Hospital, Stockholm, Sweden; 5 Center for Safety Research, KTH Royal Institute of Technology, Stockholm, Sweden; 6 Department of Clinical Physiology, National Research Council, Pisa, Italy; 7 Internal Medicine Unit, Södertälje Hospital, Södertälje, Stockholm Region Sweden

**Keywords:** Empagliflozin, Insulin resistance, Myocardial infarction, Dysglycaemia, Cardiac magnetic resonance

## Abstract

**Background:**

Empagliflozin reduces the risk of cardiovascular disease (CVD) in patients with type 2 diabetes (T2DM) and high cardiovascular risk via mechanisms which have not been fully explained. The mechanisms of such benefit have not been fully understood, and whether empagliflozin can be safely administered as first-line treatment in patients with CVD at the initial stages of glycaemic perturbations remains to be established. We investigated the effects of empagliflozin on insulin resistance, insulin sensitivity and β-cell function indexes in patients with a recent acute coronary event and newly detected dysglycaemia, i.e., impaired glucose tolerance (IGT) or T2DM.

**Methods:**

Forty-two patients (mean age 67.5 years, 19% females) with a recent myocardial infarction (n = 36) or unstable angina (n = 6) and newly detected dysglycaemia were randomized to either empagliflozin 25 mg daily (n = 20) or placebo (n = 22). Patients were investigated with stress-perfusion cardiac magnetic resonance imaging before randomization, 7 months after the start of study drug and 3 months following its cessation. Indexes of insulin resistance, sensitivity and β-cell function were calculated based on glucose and insulin values from 2-hour oral glucose tolerance tests (OGTT) and fasting C-peptide. The differences in glucose, insulin, C-peptide, mannose levels and indexes between the two groups were computed by repeated measures ANOVA including an interaction term between the treatment allocation and the time of visit.

**Results:**

After 7 months, empagliflozin significantly decreased glucose and insulin values during the OGTT, whereas C-peptide, mannose and HbA1c did not differ. Empagliflozin significantly improved insulin sensitivity indexes but did not impact insulin resistance and β-cell function. After cessation of the drug, all indexes returned to initial levels. Insulin sensitivity indexes were inversely correlated with left ventricular mass at baseline.

**Conclusions:**

Empagliflozin improved insulin sensitivity indexes in patients with a recent coronary event and drug naïve dysglycaemia. These findings support the safe use of empagliflozin as first-line glucose-lowering treatment in patients at very high cardiovascular risk with newly diagnosed dysglycaemia.

**Trial registration number:**

EudraCT number 2015-004571-73.

**Supplementary Information:**

The online version contains supplementary material available at 10.1186/s12933-023-01950-0.

## Background

Increased insulin resistance and impaired β-cell function appear before the onset of overt type 2 diabetes (T2DM) and are already present in states preceding diabetes such as impaired glucose tolerance (IGT) [1]. By inducing oxidative stress, chronic inflammation, and endothelial dysfunction, insulin resistance plays a crucial role in the pathogenesis of atherosclerosis thereby contributing to the increased cardiovascular (CV) risk [2]. Studies testing the efficacy of insulin-sensitizing strategies, such as lifestyle changes and thiazolidinediones, for ameliorating CV outcomes in people with insulin resistance have shown positive effects [3].

Recently large randomised controlled studies have proven that sodium-glucose co-transporter 2 inhibitors (SGLT2i) protect against CV events, especially heart failure, and mortality in patients with T2DM and high CV risk or heart failure, not only through their favourable impact on traditional, atherogenic CV risk factors (e.g., glycaemia, body weight, blood pressure) but possibly also by improving myocardial function and metabolism [4, 5]. This effect has been suggested to partly be attributed to improvement of insulin resistance in the failing heart [6] and in patients with T2DM [7]. Moreover, SGLT2 inhibition has been suggested to improve pancreatic β-cell function in patients with T2DM [8]. Whether metabolic and cardiac advantages may be promoted even in earlier stages of dysglycaemia in patients without heart failure are limited [9, 10].

The present objectives were to (i) investigate the effects of the SGLT2i empagliflozin on insulin resistance/sensitivity and β-cell function in patients with a recent acute coronary event and newly detected dysglycaemia, i.e., IGT or T2DM; (ii) determine whether this effect, if present, is associated with an improvement in myocardial performance and structure; (iii) investigate whether the potential impact of empagliflozin is long-lasting after cessation of the drug.

**Table 1:** Baseline characteristics of the trial cohort. Data presented are numbers (%) or mean (standard deviation) for normally distributed variables and median (Q1-Q3) for skewed variables

Variable	Empagliflozin (n = 20)	Placebo (n = 22)	Missing
Age (years)	67 (8)	68 (8)	0
Male sex	16 (80%)	18 (82%)	0
Waist circumference (cm)	100 (95, 105)	102 (95, 108)	6
BMI (kg/m^2^)	27.0 (4.1)	27.1 (4.2)	0
**Medical history**			
Index event (MI/UA)	17/3	19/3	
Prior TIA/Stroke	2 (10)	0 (0)	0
Peripheral artery disease	1 (5)	0 (0)	0
Heart failure	1 (5)	0 (0)	0
Known family history of CVD**	5 (26)	9 (45)	3
Known family history of T2DM**	5 (28)	6 (29)	3
**Glycaemic group**			
IGT	11 (55%)	14 (64%)	0
T2DM	9 (45%)	8 (36%)	
**Smoking habits**			0
Current	1 (5)	7(32)	
Previous (> 1 month)	14 (70)	12 (55)	
Never	5 (25)	3 (14)	
**Blood pressure (mmHg)**			
Systolic	130 (16)	131 (16)	0
Diastolic	80 (74, 85)	80 (77, 85)	0
**Laboratory values**			
LDL-C (mmol/L)	1.43 (0.35)	1.43 (0.59)	1
HDL-C (mmol/L)	1.24 (0.34)	1.18 (0.38)	1
Creatinine (µmol/L)	85.9 (15.6)	81.1 (18.4)	1
eGFR (ml/min/1.73 m^2^)	68.2 (12.6)	72.9 (14.0)	2
Haemoglobin (g/L)	141 (135, 150)	142 (135, 149)	1
Troponin (ng/L)	11.0 (9.0, 14.0)	11.5 (10.0, 21.0)	1
Triglycerides (mmol/L)	1.0 (0.9, 1.4)	1.2 (1.0, 1.4)	1
hs-CRP (mg/L)	1.1 (0.6, 1.7)	1.1 (0.7, 1.4)	1
NT-proBNP (ng/L)	143 (72, 514)	156 (62, 236)	1
FPG (mmol/L)	6.2 (6.0, 7.2)	6.3 (6.0, 6.7)	0
2 h-PG (mmol/L)	10.7 (8.7, 12.2)	9.7 (8.6, 12.3)	1
HbA1c (mmol/mol)	41 (39, 45)	42 (40, 47)	2
Mannose (µmol/L)	92.7 (82.3, 97.6)	86.1 (76.3, 90.1)	4
**Pharmacological treatment**			
ACE inhibitors/ARBs	17 (85)	18 (82)	0
Beta blockers	17 (85)	21 (95)	0
Calcium channel blockers	5 (25)	4 (18)	0
Diuretics	7 (35)	3 (14)	0
Statins	20 (100)	21 (96)	0
Aspirin	19 (95)	21 (95)	0

** Defined as a close relative with CVD or T2DM at < 60 years of age and based on self-reported information in standardized questionnaires

MI, myocardial infarction; UA, unstable angina; TIA, transitory ischaemic attack; IGT, impaired glucose tolerance; T2DM, type 2 diabetes; LDL-C, low-density lipoprotein; eGFR, estimated glomerular filtration rate; hsCRP, high-sensitivity C-reactive protein; BNP, brain natriuretic peptide; HbA1c, glycated haemoglobin A1c; ACE, angiotensin converting enzyme; ARB, angiotensin receptor blocker

## Methods

SOdium-glucose CO-transporter inhibition in patients with newly detected Glucose Abnormalities and a recent Myocardial Infarction (SOCOGAMI) was a randomized, double blind, placebo-controlled trial conducted at the Cardiology Unit, Department of Medicine Solna, Karolinska Institutet, Stockholm, Sweden, investigating whether empagliflozin may have beneficial effects on the myocardial function in patients with a recent acute coronary syndrome and newly detected IGT and T2DM. The trial protocol has been previously described in detail [11]. The primary endpoints were (1) changes in cardiovascular magnetic resonance (CMR) measures and (2) variations in beta-cell function estimates. The present pre-specified analysis focuses on the second of these primary endpoints, whereas the results regarding CMR measures have been published elsewhere [11]. Adult patients with a recent acute myocardial infarction (AMI) or unstable angina pectoris (< 6 months) according to joint European and American recommendations [12] and newly detected IGT or T2DM according to the World Health Organization (WHO) criteria [13] confirmed by two screening oral glucose tolerance tests (OGTT), were recruited. Exclusion criteria were known diabetes, estimated glomerular filtration rate (eGFR) < 30 ml/min/1.73m^2^, contraindications to cardiac magnetic resonance (CMR) imaging, contraindications or known intolerance to intravenous adenosine, severe concomitant disease, planned coronary revascularization procedures, congestive heart failure with New York Heart Association (NYHA) class III-IV and women of childbearing potential [11].

**Fig. 1:**
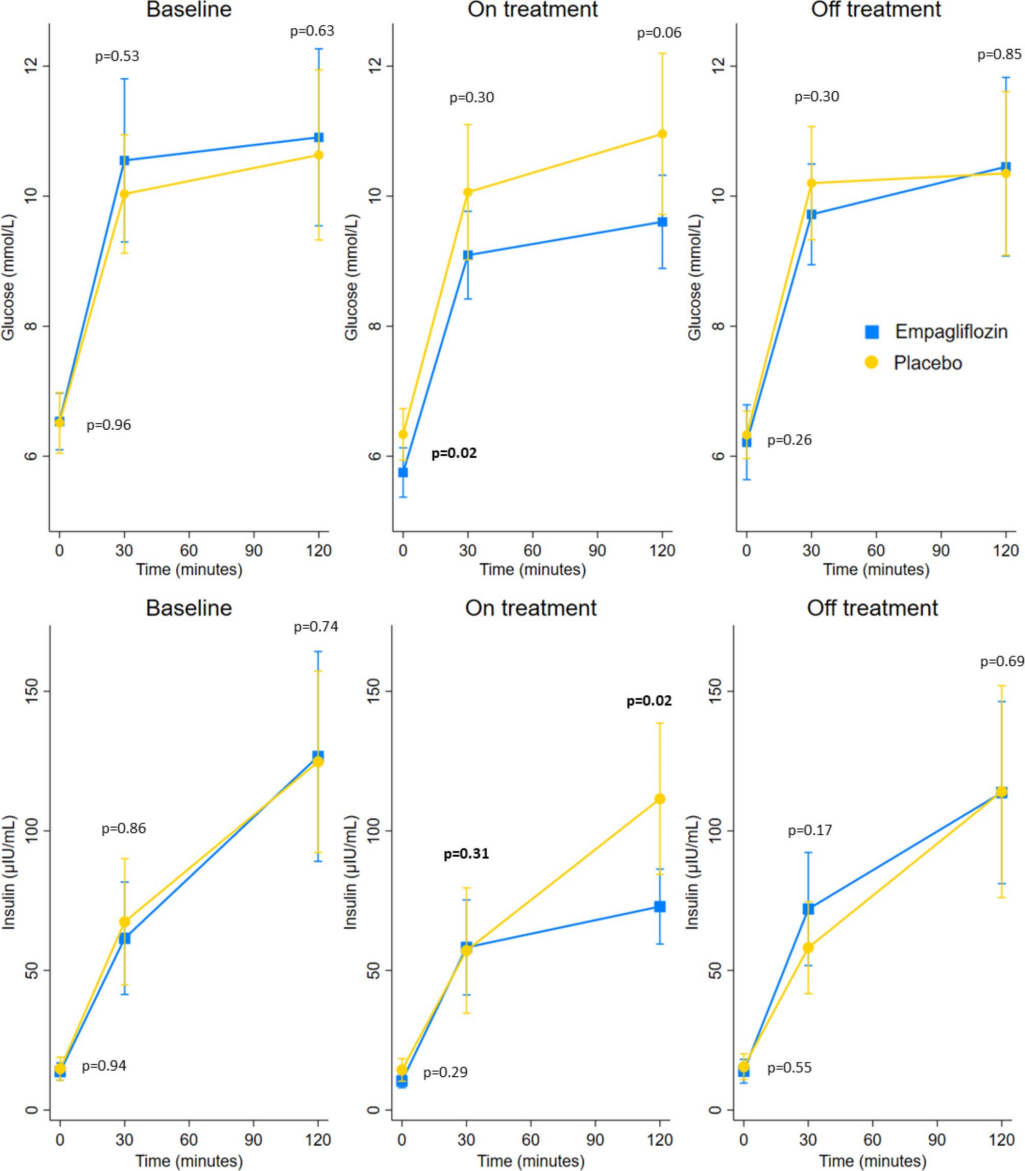
Changes in insulin and glucose levels during the OGTT at the three different time points. P-values are by Mann-Whitney U-test

### Study protocol

Medical history, concomitant therapies, smoking status, and working status were collected during the baseline visit. A physical examination including body mass index (BMI), waist circumference, blood pressure and heart rate was performed. Blood samples were collected after 12 h of fasting. Subsequently, every patient proceeded with a 2-hour oral glucose tolerance test (OGTT). A CMR imaging was performed after completion of the OGTT. Thereafter, patients were randomized to either 25 mg of empagliflozin once daily or placebo. All patients received a diary together with equipment for self-monitoring of blood glucose and were scheduled for follow-up at the outpatient clinic one and three months later. Seven months after randomization all investigations performed during the baseline visit were repeated. Thereafter, the study drug was stopped and three months later (i.e., ten months after randomization) all patients returned for a final visit including all investigations; this off-drug period was carried out to investigate whether the potential impact of the drug was long-lasting.

A safety evaluation during the time on study drug was conducted on all patients.

**Fig. 2:**
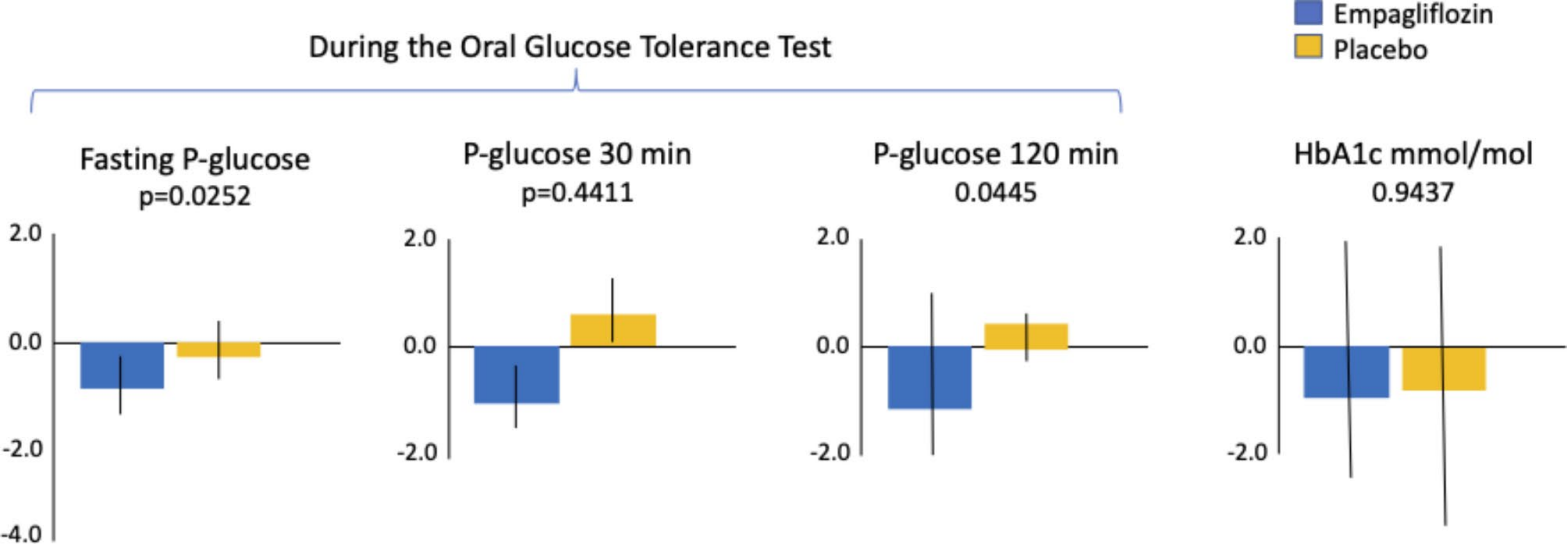
Changes in plasma glucose levels and HbA1c from baseline to after seven months on study drug. Legend: HbA1c: glycated haemoglobin A1c

### Methods

*2-hour OGTT* was performed by administration of 75 g of glucose in 200 ml water following an overnight fast of 12 h. A plasma glucose curve was obtained with values at baseline, after 30 min and after 120 min by HemoCue® Glucose 201 RT (HC201RT) equipment [14].

**Table 2:** Indexes of β-cell function, insulin resistance and insulin sensitivity by treatment group over the three time points of the trial. Values are mean (standard deviation) for normally distributed variables and median (Q1-Q3) for skewed variables. P_I(0−7)_ and P_I(7−10)_ are P values for interaction between treatment allocation and time of visit in the repeated measures ANOVA model, for the pairwise comparisons between 0 and seven months and seven and ten months respectively, corrected for multiple testing by Bonferroni method. P_I overall_ is P for interaction between treatment allocation and time of visit in the whole ANOVA model

	Baseline		7 months			10 months			
	**Empagliflozin**	**Placebo**	**Empagliflozin**	**Placebo**	P_I(0−7)_	**Empagliflozin**	**Placebo**	P_I(7−10)_	P_I overall_
**Indexes of β-cell function**									
Insulinogenic index	84.8 (58.8, 100.0)	72.4 (48.0, 111.7)	64.2 (57.1, 98.3)	57.9 (48.8, 75.2)	0.47	107.3 (78.3, 129.4)	65.1 (47.8, 88.3)	0.33	0.69
First phase Stumvoll	800.2 (600.0)	921.8 (596.0)	897.1 (460.9)	795.1 (617.3)	0.55	1066.0 (525.5)	844.0 (503.9)	0.55	0.55
s phase Stumvoll	242.0 (130.7)	265.5 (137.0)	253.5 (104.0)	237.4 (140.9)	0.47	297.3 (121.9)	248.9 (117.8)	0.47	0.47
C-peptide index	2.82 (2.40, 3.39)	2.85 (2.03, 3.44)	2.58 (2.09, 3.69)	2.56 (1.90, 3.64)	0.33	2.69 (2.10, 3.79)	2.98 (2.02, 3.44)	0.33	0.40
totAUCIGI	45.5 (23.3)	51.1 (24.6)	39.2 (15.2)	42.3 (19.8)	0.18	51.9 (19.7)	43.3 (21.11)	0.67	0.18
HOMA2-β (mU/l, mmol/l)	72.6 (61.9, 94.2)	73.5 (58.6, 101.8)	70.5 (50.5, 117.0)	76.5 (47.5, 123.5)	0.55	83.5 (60.0, 92.4)	88.4 (54.2, 104.2)	0.55	0.55
Proinsulin/Insulin	0.18 (0.07)	0.17 (0.06)	0.17 (0.07)	0.16 (0.06)	0.71	-	-	-	0.71
**Indexes of insulin resistance**									
HOMA-IR (traditional)	4.0 (1.8)	4.3 (3.0)	2.6 (1.3)	4.0 (2.5)	0.07	4.0 (2.8)	4.5 (3.2)	0.011	0.19
HOMA2-IR	1.5 (1.1, 1.8)	1.5 (1.0, 2.1)	1.4 (0.6, 1.7)	1.4 (0.9, 2.3)	0.28	1.5 (1.0, 1.8)	1.6 (1.0, 2.1)	0.03	0.20
VAI	1.39 (1.09, 1.63)	1.64 (1.16, 2.44)	1.08 (0.82, 1.82)	1.63 (1.15, 2.02)	0.82	1.12 (0.77, 1.90)	1.45 (1.30, 2.22)	0.82	0.82
LAP	37.2 (26.9, 51.4)	46.8 (32.7, 62.7)	36.1 (19.4, 48.0)	42.1 (30.2, 54.0)	0.95	33.84 (28.5, 50.4)	44.1 (35.3, 59.3)	0.95	0.95
**Indexes of insulin sensitivity**									
QUICKI	0.32 (0.3, 0.33)	0.32 (0.30, 0.34)	0.33 (0.32, 0.36)	0.32 (0.30, 0.33)	**0.0001**	0.32 (0.31, 0.34)	0.31 (0.30, 0.33)	**0.004**	**0.01**
HOMA2-S	66.8 (54.6, 88.3)	68.5 (47.7, 101.3)	71.1 (59.0, 171.3)	70.5 (44.3, 108.5)	**0.001**	68.9 (56.9, 96.8)	64.6 (47.1, 99.0)	**0.014**	**0.03**
Stumvoll MCR_120_	9.26 (8.43, 9.67)	9.14 (8.27, 9.61)	9.68 (8.46, 10.04)	9.06 (8.22, 9.55)	**0.0001**	9.22 (8.46, 10.04)	9.06 (8.22, 9–55)	**0.004**	**0.003**
Matsuda Index	2.22 (1.48–3.15)	2.53 (1.59–3.02)	2.78 (2.29–4.36)	3.13 (2.13–3.59)	0.14	2.32 (1.82–2.51)	3.04 (1.02–4.00)	0.34	**0.05**

AUCIGI: area under the curve of insulinogenic index; HOMA-IR: homeostatic model assessment for insulin resistance; ISI: insulin sensitivity index; LAP: lipid accumulation product; MCR: Metabolic clearance rate of glucose; QUICKI: quantitative insulin sensitivity check index; VAI: visceral adiposity index.

*Proinsulin* was measured with a non-competitive sandwich-enzyme-linked immunosorbent assay (ELISA) with photometric detection (Mecordia; reference interval 3.3–28 pmol/L).

*Insulin* was measured with an electrochemical luminescence immunoassay (ECLIA) (Roche, reference interval 2.0–25 mU/L for adults in the fasting state).

*C-peptide* was measured with an ECLIA (Roche, reference interval 0.5–1 nmol/L in the fasting state).

Plasma insulin was measured during all OGTT time points while C-peptide (from all three visits) and proinsulin (from the two first visits) was only measured on fasting samples.

*β-cell function and insulin resistance indexes* were calculated according to the equations displayed in Supplemental Table 1. The insulinogenic index and the area under the curve of insulin (AUCIns) divided by the area under the curve of glucose (AUCGlu) were calculated during the 0- to 30-min (early) and 0- to 120-min (total) time of the OGTT, respectively. AUCs were calculated in accordance with the trapezoidal rule applied to the insulin and glucose curves during the OGTT. The Matsuda Index was calculated from glucose and insulin levels at 0, 30, and 120 min of the OGTT, adjusting for urinary excretion glucose levels in patients on treatment with empagliflozin (http://mmatsuda.diabetes-smc.jp/MIndex.html).

**Table 3:** Spearman correlations between insulin sensitivity indexes and CMR parameters at baseline and their differences between baseline and seven months, in the empagliflozin and in the placebo groups

**Empagliflozin**												
	LVMi	LVESVi	LVEF	LVEDVi	LVSVi	ECV	∆LVMi	∆LVESVi	∆LVEF	∆LVEDVi	∆LVSVi	∆ECV
Stumvoll MCR_120_	− 0.86*	0.27	− 0.17	0.32	− 0.36	− 0.02						
HOMA2S	− 0.65*	0.51	− 0.32	0.63*	− 0.05	− 0.08						
QUICKI	− 0.56*	0.43	− 0.23	0.53	− 0.02	− 0.21						
∆Stumvoll MCR_120_							− 0.35	0.28	− 0.01	0.49	0.32	0.18
∆HOMA2S							− 0.13	0.80*	− 0.40	0.79*	0.12	0.04
∆QUICKI							− 0.27	0.81*	− 0.41	0.82*	0.14	− 0.06
**Placebo**												
	LVMi	LVESVi	LVEF	LVEDVi	LVSVi	ECV	∆LVMi	∆LVESVi	∆LVEF	∆LVEDVi	∆LVSVi	∆ECV
Stumvoll MCR_120_	− 0.78*	0.35	− 0.36	0.26	− 0.22	0.18						
HOMA2S	− 0.48*	0.60*	− 0.53*	0.50*	0.06	0.31						
QUICKI	0.41	0.56*	− 0.46*	0.48*	0.11	0.21						
∆Stumvoll MCR_120_							− 0.59*	− 0.09	0.13	0.003	− 0.02	− 0.12
∆HOMA2S							− 0.08	− 0.24	0.33	0.12	0.21	0.02
∆QUICKI							0.03	− 0.36	0.36	− 0.02	0.15	0.14

* p ≤ 0.05

HOMA, Homeostasis model assessment; ISI, insulin sensitivity index; Stumvoll MCR_120_, metabolic clearance rate QUICKI, quantitative insulin sensitivity check index

*Fasting plasma mannose* concentrations were obtained by means of high-performance liquid chromatography coupled to tandem mass spectrometry (HPLC-MS-MS) at the Mass Spectrometry laboratory in Pisa, Italy [15].

CMR: The CMR procedure was previously described in detail [11]. In brief, patients underwent CMR at 1.5 T (Siemens Aera, Siemens Healthineers, Erlangen, Germany) including first pass perfusion imaging using an intravenous contrast agent (Gadobutrol, Gadovist, Bayer AB, Solna, Sweden), both at stress (by an adenosine infusion of 140 microg/min/kg body weight [Adenosine, Life Medical AB, Stockholm, Sweden]), and at rest. Left ventricular volumes, systolic function, stroke volume, and mass were assessed by cine, steady-state free precession imaging. Late gadolinium enhancement (LGE) was used to identify infarcted myocardium [16].

**Fig. 3:**
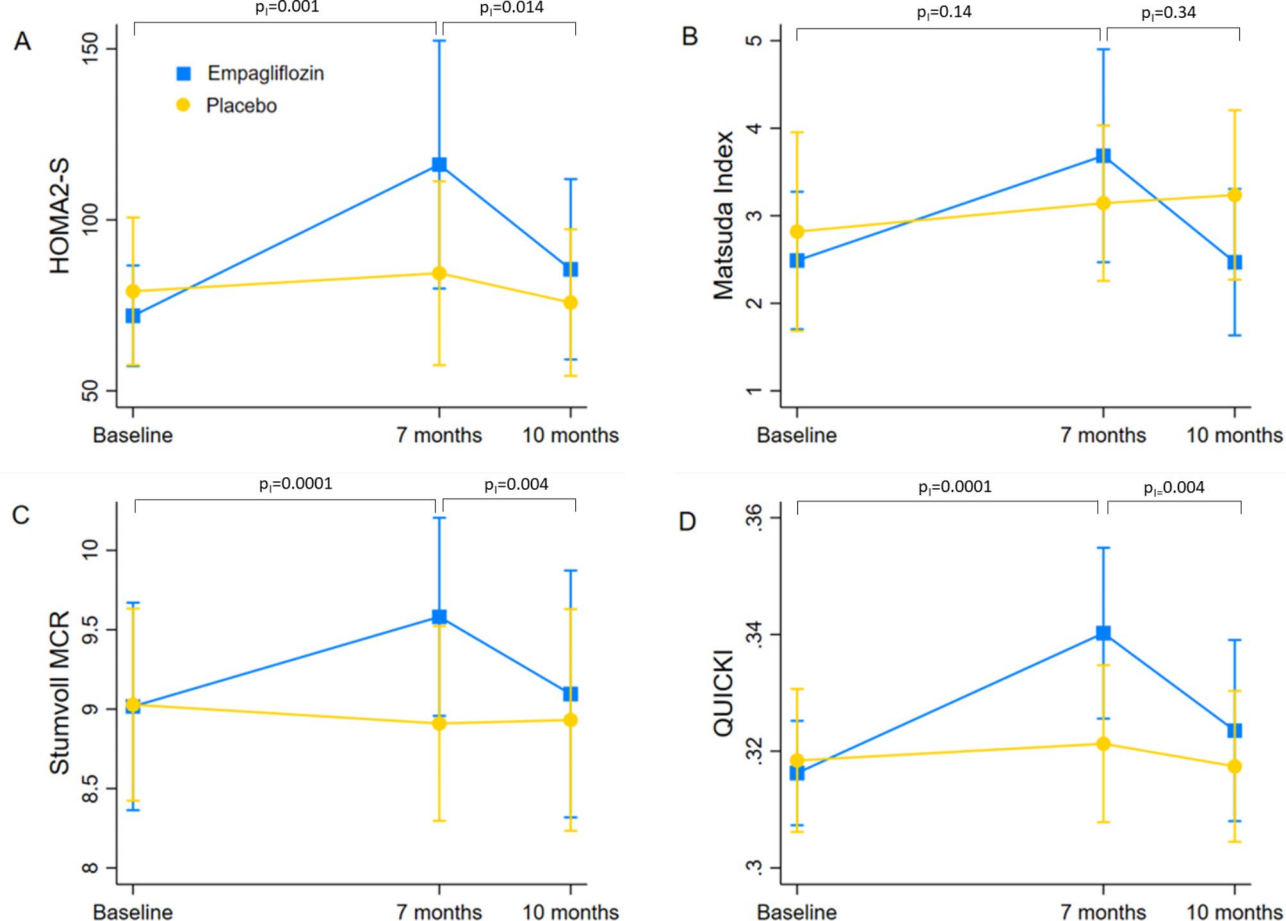
Changes in insulin sensitivity indexes at baseline, after seven months and ten months. P_I(0−7)_ and P_I(7−10)_ are P values for interaction between treatment allocation and time of visit in the repeated measures ANOVA model, for the pairwise comparisons between 0 and seven months and seven and ten months respectively, corrected for multiple testing by Bonferroni method. P_I overall_ is P for interaction between treatment allocation and time of visit in the whole ANOVA model. Legend: HOMA: Homeostatic Model Assessment; QUICKI: quantitative insulin sensitivity check index; MCR: metabolic clearance rate

All CMR images analyses were performed using Segment CMR [17].

### Statistics

#### Power calculation

A sample of 60 patients was required to detect an assumed difference in LV end-diastolic volume of 15% with a significance level of 5%, and a coefficient of variation of at most 80% and a power of 80%. An interim analysis, blinded for the investigators, was performed after the recruitment of 40 patients, who had passed the visit after seven months. This revealed that further patient inclusions would not be meaningful, which was the reason for stopping further inclusions.

Baseline characteristics of patients receiving empagliflozin vs. placebo are presented as mean ± standard deviation (SD) for variables with a normal distribution or median [1st and 3rd quartile (Q1, Q3)] for variables with a skewed distribution according to the Shapiro-Wilk test. Differences in baseline characteristics were assessed by the t-test or Mann-Whitney U-test, as appropriate, for continuous variables and by the χ^2^ test for categorical variables.

Differences in indexes of insulin resistance/β-cell function across the three time points were investigated by repeated measures ANOVA, including an interaction term between the treatment allocation and the time of visit. The sphericity assumption of ANOVA models was checked with the Mauchly´s W test. Correction for multiple testing was performed by Bonferroni adjustment and the level of significance of the interaction p values was set at 0.01. The associations between the indexes whose change overtime resulted statistically significant and CMR parameters were investigated both at baseline and taking into consideration the change from baseline and seven months, separately in patients receiving empagliflozin vs. placebo.

All statistical analyses were performed using STATA/MP 17.0.

## Results

### Baseline characteristics of the study population

Forty-two patients (IGT n = 27; T2DM n = 15; AMI = 36; unstable angina = 6) fulfilled the selection criteria and were randomized to receive either empagliflozin 25 mg/day (n = 20) or placebo (n = 22). Among those randomized to empagliflozin, the proportion of patients with IGT was 60% and T2DM 40%. The corresponding proportions in the placebo group were 68% and 32% respectively. The two groups were well balanced as regards baseline characteristics (Table 1).

### Effects of empagliflozin on glycemic control, insulin, C-peptide, and mannose levels

Baseline concentrations of fasting and post-load glycaemic variables, i.e., glucose, insulin, proinsulin and C-peptide, did not differ between the two groups, while mannose levels were slightly higher in the empagliflozin group compared to placebo (92.7 vs. 86.1 µmol/L, p = 0.04; Supplemental Table 2) and were significantly higher in patients with T2DM than those with IGT (86.1 vs. 76.1 µmol/L, p = 0.02).

After seven months of treatment, fasting plasma glucose (FPG), 2-hour post-load glucose (2hPG) and 2-hour post-load insulin were significantly reduced by empagliflozin compared with placebo, a difference no longer visible three months after drug cessation (Figs. 1 and 2 and Supplemental Table 2). HbA1c and mannose levels at seven months did not differ between the two treatment groups and between glycaemic categories (Fig. 2 and Supplemental Figure S1).

### Effects of empagliflozin on indexes of insulin resistance/sensitivity and β-cell function

Table 2 summarizes the baseline, seven-month and ten-month values for several indexes of insulin resistance/sensitivity and β-cell function in the two study arms. The p value for interaction between treatment allocation and time of visit in the repeated measures ANOVA model was consistently statistically significant for indexes of insulin sensitivity, i.e., Homeostatic Model Assessment (HOMA2-S), Stumvoll metabolic clearance rate (MCR_120_), HOMA2-S and quantitative insulin sensitivity check index (QUICKI). The Matsuda index also approached significance, with a p value for interaction of 0.052. As shown by the pairwise comparisons between the three time points (Table 2; Fig. 3), the differences in HOMA2-S, Stumvoll MCR_120_ and QUICKI from baseline to seven months are attributable to a significant increase of these indexes in the treatment group. This effect disappears three months after drug cessation, with the indexes returning to their initial values at ten months.

As previously reported, there was no effect of empagliflozin on the CMR variables [11]. When investigating simple correlations between insulin sensitivity indexes and CMR parameters, Stumvoll MCR and HOMA2-S were consistently inversely correlated with left ventricular mass. In the empagliflozin group, differences from baseline to seven months in HOMA2-S and QUICKI were positively correlated with differences in end-systolic and end-diastolic left ventricular volume (Table 2).

No unforeseen safety concerns were identified, as previously reported in detail [11].

## Discussion

The main finding in this investigation of patients with a recent acute coronary syndrome and newly diagnosed dysglycaemia was that (i) seven months of treatment with the SGLT2i empagliflozin improved insulin sensitivity indexes; (ii) changes in those insulin sensitivity indexes were correlated with changes in cardiac volumes as measured by CMR after seven months of empagliflozin therapy; (iii) the impact of empagliflozin was not long-lasting as revealed by the findings from three months after cessation of the drug.

Chronic hyperglycaemia impairs both insulin sensitivity and pancreatic insulin secretion, initiating a vicious cycle that gradually increases insulin resistance and deteriorates β-cell function [18]. This process, commonly known as glucotoxicity, ultimately leads to β-cell failure, decreased insulin secretory capacity and overt T2DM [18]. Several preclinical and clinical studies reported that SGLT2i have a positive impact on both insulin resistance and β-cell function, suggesting that they may be able to stop or retard the progression from hyperglycaemia to diabetes by reversing the glucotoxic effect of chronic hyperglycaemia on the pancreatic β-cells (8, 24–26). In the present study, empagliflozin significantly lowered FPG, 2hPG and 2 h-insulin levels after seven months of treatment compared to placebo. This reduction in plasma glucose and insulin concentrations resulted in an improvement in insulin sensitivity, as measured with HOMA2-S, QUICKI, and MCR Stumvoll indexes, whereas no significant changes were observed in β-cell function. In fact, SGLT2i may mainly affect insulin sensitivity indexes because of lower glucose and insulin levels induced by increased glucose urinary excretion, as already shown in studies on patients with established T2DM [7, 19]. The present findings extend these results by including drug-naïve patients with newly detected T2DM or IGT, whose insulin resistance and β-cell function are presumably less deteriorated. As a confirmation, HbA1c levels in our population were much lower than in the aforementioned studies, and indeed HbA1c was not influenced by empagliflozin treatment in SOCOGAMI.

Our findings partly contradict the results of previous studies. A placebo-controlled clinical study conducted by Merovci et al., reported that two-weeks treatment with dapagliflozin in patients with uncontrolled T2DM not only improved insulin sensitivity (based on euglycaemic clamp), but also led to an improved β-cell function, calculated as the ratio between the change in C-peptide and the change in glucose values during a 2 h OGTT [20]. Similar results have been observed in other populations with T2DM, including a Japanese cohort where ipragliflozin significantly improved the β-cell function assessed by an OGTT-derived disposition index and an Italian study where empagliflozin enhanced β-Cell glucose sensitivity and increased insulin clearance [7, 21]. The two main factors possibly explaining these differences are the use of different indexes to express β-cell function and the inclusion of patients with established, long-term T2DM and a more impaired β-cell function. Accordingly, a recent placebo-controlled clinical trial in patients with IGT did not find any changes in insulin secretion following six-weeks treatment with 10 mg/day of dapagliflozin, despite an increase in insulin sensitivity [9], confirming previous findings in rodents [22]. The deterioration of β-cell function at the early stages of dysglycaemia might be subclinical and driven by additional factors than those reflected by the glycaemic profile, such as lipotoxicity and inflammation [23].

The fact that plasma mannose, an emerging, sensitive marker of insulin resistance and cardiovascular disease [24, 25], was not affected by empagliflozin treatment could be due to the presence of significant differences already at baseline not only between the two treatment groups, but also according to the glycaemic groups, therefore further restricting the sample size.

The specific mechanisms of the beneficial effects of SGLT2i on cardiovascular disease remain to be defined [26]. It has been suggested that relieved insulin resistance might play a role [27]. In SOCOGAMI, treatment with empagliflozin did not influence any CMR parameter [11], therefore the significant correlations we found between indexes of insulin sensitivity and left ventricular mass should be taken with caution. Nevertheless, there is evidence that insulin-sensitizing interventions reduced cardiovascular morbidity and mortality in insulin-resistant patients, possibly because of direct effects on myocardial function and structure [28–30]. In the EMPA-HEART CardioLink-6 Randomized Clinical Trial, treatment with empagliflozin in patients with T2DM and coronary artery disease resulted in a significant reduction of left ventricular mass [31]. In contrast, we found a strong inverse correlation between insulin sensitivity indexes and left ventricular mass in both groups at baseline, and the improvement of insulin sensitivity observed after seven months of empagliflozin was not significantly associated with any mass variation of the left ventricle. A bigger sample size and the inclusion of patients with long-standing T2DM and with more increased cardiac mass at baseline, might partly explain these discrepant results. In SOCOGAMI, 60% of patients had IGT, which is a prognostically unfavorable condition by itself [32]. However, to date there is no uniformly recommended pharmacological intervention in such patients. These issues are not insignificant in very high-risk populations who have already suffered a coronary event, as those included in SOCOGAMI. Empagliflozin might be a treatment of choice in patients with IGT at high cardiovascular risk, and its good safety profile is already confirmed by trials conducted in populations with heart failure and without overt diabetes [33]. Even in patients with T2DM who had a coronary event, where metformin since long has been recommended as the first-line therapy, it might be reasonable to prioritize an SGLT2i, although there are no current direct comparisons between metformin and SGLT2i as regards their insulin-sensitizing effects. Longitudinal studies investigating cardiovascular outcomes in IGT patients are needed to strengthen these assumptions.

### Strengths and weaknesses

Major strengths of this study include its double-blind, randomized design and investigations at three different time points, including after treatment withdrawal, which further confirms that the observed effects are attributable to empagliflozin. Several validated indexes were described, permitting a detailed assessment of the effects of empagliflozin on different processes involved in glucose homeostasis, as well as on other variables associated with insulin resistance, e.g., mannose levels and lipid indexes. Another strength is the use of CMR to examine myocardial performance and structure.

This study also has some limitations. The sample size was relatively limited, and OGTT-derived surrogates were used for measuring insulin resistance/sensitivity and β-cell function. An OGTT is widely used to diagnose diabetes and represents the only accepted method to detect IGT [34], but it is less reproducible than an intravenous GTT due to variations in glucose absorption and incretin hormone secretion. In addition, both peripheral and hepatic insulin sensitivity are typically included in OGTT-derived surrogates [35]. Therefore, the application of surrogate indexes may make it difficult to assess the direct metabolic actions of insulin. As previously underlined, patients included in SOCOGAMI did not have a compromised left ventricular function, thus the cardiac benefits of empagliflozin might have been hard to detect.

Finally, it cannot be excluded that the modest but significantly different decrease in BMI in the empagliflozin-treated group (–0.5 ± 0.8) compared to that observed in the placebo group (–0.3 ± 0.8) may have, at least in part, contributed to the improvement in insulin sensitivity.

## Conclusion

Empagliflozin improved insulin sensitivity indexes in patients with a recent myocardial infarction or unstable angina, without heart failure, and with newly detected, drug naïve diabetes or impaired glucose tolerance.

### Electronic supplementary material

Below is the link to the electronic supplementary material.

Supplementary Material 1

## Data Availability

Data supporting this study are available from the senior author upon reasonable request.
