# Supplementary material for: Empagliflozin improves insulin sensitivity in patients with recent acute coronary syndrome and newly detected dysglycaemia: Experiences from the randomized, controlled SOCOGAMI trial
Source: Cardiovasc Diabetol. 2023 Aug 11;22:208. doi: 10.1186/s12933-023-01950-0 (PMC10422806; doi:10.1186/s12933-023-01950-0)

Supplemental Material

Supplemental Table 1. Indexes of insulin resistance and β-cell function

Name	*Formula*	*Ref*
**Indexes of insulin resistance/sensitivity**		
HOMA-IR	$\frac{G0 \times I0}{22.5}$	(1)
HOMA2-IR	https://www.dtu.ox.ac.uk/homacalculator/ *	(2)
QUICKI	$\frac{1}{\log10(I0) + \log10(G0)}$	(3)
HOMA2-S	https://www.dtu.ox.ac.uk/homacalculator/ *	(2)
Stumvoll MCR_{120}	$19.240 - 0.281 \times BMI - 0.00498 \times I120 - 0.333 \times G120$	(4)
Matsuda ISI	$\frac{10{,}000}{\sqrt{(G0 \times I0) \times (G120 \times I120)}}$	(5, 6)
VAI	Men: $(\frac{WC}{39.68+(1.88 \text{ x } BMI)}) \text{ x } (\frac{TG}{1.03}) \text{ x } (\frac{1.31}{HDL})$<br>Women: $(\frac{WC}{36.58+(1.89 \text{ x } BMI)}) \text{ x } (\frac{TG}{0.81}) \text{ x } (\frac{1.52}{HDL})$	(7, 8)
LAP	Men: (WC - 65) x (TG)<br>Women: (WC- 58) x (TG)	(7)
**Indexes of insulin secretion/ β-cell function**		
Insulinogenic index	(I30-I0) / (G30-G0)	(9, 10)
First phase Stumvoll	$1{,}283 + 1.829 \times I30 - 138.7 \times G30 + 3.772 \times I0$	(11)
Second phase Stumvoll	$286 + 0.416 \times I30 - 25.94 \times G30 + 0.926 \times I0$	
Fasting C-peptide index (CPI) (ng/mL, mg/dL)	$\frac{Cp0}{G0} \times 100$	(12)
Total $AUC_{Ins/Glu}$	$\frac{\text{AUC Insulin}}{\text{AUC Glucose}}$	(13)
HOMA2-β	https://www.dtu.ox.ac.uk/homacalculator/ *	(2)
Proinsulin/Insulin	$\frac{\text{Proinsulin0}}{I0}$	
Insulin secretion sensitivity index 2 (ISSI-2)	$\frac{\text{Total AUC(Ins/Glu)}}{\text{Matsuda Index}}$	(13, 14)

HOMA-IR, QUICKI and Matsuda ISI were calculated from insulin concentrations given in mU/L. QUICKI and PCPI were calculated from glucose concentrations given in mg/dL. All other indexes were derived from the OGTT with insulin concentrations given in pmol/l, and glucose concentration given in mmol/l. VAI and LAP were calculated from triglycerides and HDL in mmol/l.
* HOMA2 Calculator version 2.2.3 (Diabetes Trials Unit, University of Oxford, Oxford, UK).

AUC, area under the curve; BMI, body mass index; MCR, metabolic clearance rate; G0, fasting plasma glucose; G120, 2-hour post-load glucose; HDL, high-density lipoprotein; HOMA-IR, homeostasis model assessment-insulin resistance; I0, fasting plasma insulin; I120, 2-hour post-load insulin; IGI, Integrated glucose-insulin; ISI, insulin sensitivity index; LAP, lipid accumulation product; Proinsulin0, fasting proinsulin; QUICKI, quantitative insulin sensitivity check index; TG= triglycerides; VAI= visceral adiposity index; WC, waist circumference.

Supplemental Table 2. Insulin, C-peptide and Proinsulin at the three different time points in empagliflozin vs placebo treated patients.

Factor	Empagliflozin (n=20)	Placebo (n=22)	P*	P_I
**Baseline**				
FPG (mmol/L)	6.2 (6.0, 7.2)	6.3 (6.0, 6.7)		
30min-PG (mmol/L)	10.3 (9.4, 11.3)	9.9 (9.1, 11.4)		
2h-PG (mmol/L)	10.7 (8.7, 12.2)	9.7 (8.6, 12.3)		
Fasting insulin (μIU/mL)	13 (9.7, 15)	12 (8.4, 18)		
30min-insulin (μIU/mL)	62 (33, 89)	58.5 (36, 87.5)		
2h-insulin (μIU/mL)	103 (77, 177)	89 (84, 195)		
Fasting proinsulin (pmol/L)	13 (9.4, 17)	10.5 (8.4, 18)		
Fasting C-peptide (ng/mL)	1.05 (0.89, 1.4)	1.05 (0.78, 1.3)		
HbA1c (mmol/mol)	41 (39, 45)	42 (40, 47)		
Fasting mannose (μmol/L)	92.7 (82.3, 97.6)	86.1 (76.3, 90.1)		
**On treatment (7 months)**				
FPG (mmol/L)	5.6 (5.3, 6.1)	6.2 (5.8, 6.9)	**0.02**	
30min-PG (mmol/L)	9.1 (8.8, 9.5)	9.4 (8.5, 11.2)	0.30	
2h-PG (mmol/L)	9.7 (8.5, 10.6)	11.0 (8.9, 12.0)	0.06	
Fasting insulin (μIU/mL)	12.5 (5, 15)	12 (8.05, 20.5)	0.29	
30min-insulin (μIU/mL)	59 (39, 64)	41 (31, 58)	0.31	
2h-insulin (μIU/mL)	68 (48, 92)	92 (64, 152)	**0.02**	
Fasting proinsulin (ng/mL)	9.6 (4.6, 13)	9.3 (6.6, 18)	0.36	
Fasting C-peptide (ng/mL)	0.95 (0.71, 1.15)	0.94 (0.78, 1.3)	0.51	
HbA1c (mmol/mol)	42 (39, 44.5)	42 (40, 45)	0.88	
Fasting mannose (μmol/L)	83.8 (75.1, 91.7)	79.5 (69.0, 94.8)	0.53	
**Off treatment (10 months)**				
FPG (mmol/L)	6.0 (5.5, 6.4)	6.2 (5.8, 6.8)	0.26	**0.039**
30min-PG (mmol/L)	9.8 (8.8, 11.0)	10.3 (9.5, 11.2)	0.30	0.73
2h-PG (mmol/L)	11.2 (8.5, 12.3)	10.2 (8.1, 12.5)	0.85	0.10
Fasting insulin (μIU/mL)	12 (8.8, 15)	13 (8.7, 18.5)	0.55	0.25
30min-insulin (μIU/mL)	65 (41, 87)	58 (34, 66)	0.17	0.66
2h-insulin (μIU/mL)	103 (68, 129)	78 (59, 178)	0.69	**0.008**
Fasting C-peptide (ng/mL)	0.98 (0.74, 1.2)	1 (0.84, 1.3)	0.78	**0.048**
HbA1c (mmol/mol)	43.5 (39, 46)	44 (39, 46)	0.95	0.99
Fasting mannose (μmol/L)	84.6 (79.5, 92.6)	85.5 (72.3, 94.0)	0.86	0.57

Data are median (Q1, Q3).
*P-value by Mann-Whitney U-test.
♦P_I = P for interaction between treatment allocation and time of visit in the repeated measures ANOVA model

Supplemental Figure 1. Mannose levels at the three time points in the empagliflozin and placebo groups.

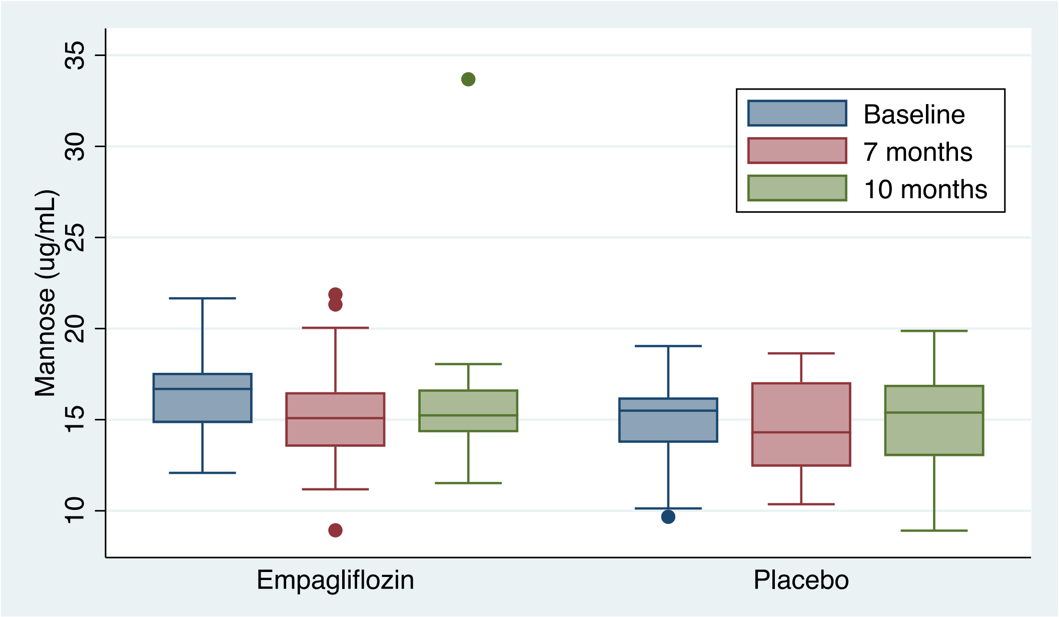

Supplement: Supplementary file 1 — Supplementary Material 1 [file 12933_2023_1950_MOESM1_ESM.pdf]
